# Epigenetic regulation in adult stem cells and cancers

**DOI:** 10.1186/2045-3701-3-41

**Published:** 2013-10-09

**Authors:** Lama Tarayrah, Xin Chen

**Affiliations:** 1 Department of Biology, The Johns Hopkins University, Baltimore, MD 21218, USA

**Keywords:** Adult stem cell, Germline stem cell, Intestinal stem cell, Hair follicle stem cell, Epigenetics, Cancer, Cancer stem cell

## Abstract

Adult stem cells maintain tissue homeostasis by their ability to both self-renew and differentiate to distinct cell types. Multiple signaling pathways have been shown to play essential roles as extrinsic cues in maintaining adult stem cell identity and activity. Recent studies also show dynamic regulation by epigenetic mechanisms as intrinsic factors in multiple adult stem cell lineages. Emerging evidence demonstrates intimate crosstalk between these two mechanisms. Misregulation of adult stem cell activity could lead to tumorigenesis, and it has been proposed that cancer stem cells may be responsible for tumor growth and metastasis. However, it is unclear whether cancer stem cells share commonalities with normal adult stem cells. In this review, we will focus on recent discoveries of epigenetic regulation in multiple adult stem cell lineages. We will also discuss how epigenetic mechanisms regulate cancer stem cell activity and probe the common and different features between cancer stem cells and normal adult stem cells.

## Introduction

Adult stem cells are defined as cells that have two central properties: self-renewal and differentiation. Many types of adult stem cells have the remarkable ability to undergo asymmetric mitotic divisions that produce two distinct daughter cells. Alternatively, they undergo symmetric divisions in a stochastic manner to produce more stem cells and differentiating cells. One daughter maintains the stem cell properties, while the other differentiates to replenish specialized cell types. The ability of adult stem cell derivatives to divide and differentiate to replace damaged tissues provides the body with an internal repair system.

Previous studies on adult stem cells have focused on understanding how extrinsic signaling pathways regulate proper stem cell functions. In addition, recent evidence shows that intrinsic factors, such as chromatin structure of stem cells, play important roles in regulating stem cell identity and activity. Epigenetic mechanisms alter the chromatin state of genes without altering their primary DNA sequences. Three major epigenetic mechanisms are known to cooperate in stem cells: nucleosome repositioning driven by chromatin remodeling factors, DNA methylation, and post-translational modifications of histones, including methylation, phosphorylation, acetylation, ubiquitination, and sumoylation [1]. Together, these mechanisms may establish a distinct epigenetic state that leads to a unique gene expression pattern in stem cells [2]. Perturbations of these epigenetic mechanisms may lead to premature differentiation or continuous self-renewal/proliferation of stem cells, a hallmark of cancer.

The relationship between carcinogenesis and changes in specific gene expression or genome stability has been well documented [3-6]. Two major epigenetic mechanisms, DNA methylation and post-translational modifications of histones, have been shown to contribute to the initiation and progression of cancers [7-11]. Accumulation of aberrant genetic mutations or abnormal epigenetic profiles could lead to tumor initiation in adult stem cell lineages [12-14]. For example, using the lineage-tracing method, studies in mice have shown that aged intestinal stem cells (ISCs) accumulate cancer-causing mutations [13,15]. However, while most studies characterize epigenetic alterations in cancers using cancer cell lines or the entire tumor, cells within a tumor display a wide degree of heterogeneity, and not all of them have the ability to initiate and sustain a tumor [16,17]. Recently, it has been proposed that a small population of cancer cells, termed cancer stem cells (CSCs), is distinct from other tumor cells and has the capacity to drive tumor initiation and growth. By definition, CSCs are a subset of tumor cells that have the capacity to self-renew, the potential to develop into any other cells in the tumor, and the proliferative ability to drive continued tumor expansion [18]. In the past decade, CSCs were found to exist in a wide range of solid tumors [19-24]. CSCs are currently being targeted in cancer treatments; however, they are relatively resistant to a variety of chemo- and radiotherapy [25]. Therefore, a better understanding of the biology of CSCs, including epigenetic alterations that affect their function, is essential for developing effective cancer therapies. On the other hand, the existence of CSCs raises the concern that conclusions based on studies using entire tumors might not apply to CSCs.

In this review, we will start by discussing the most recent discoveries in epigenetic regulation of normal adult stem cell lineages in multiple stem cell systems and across several different model organisms. We will then take up the question of epigenetic regulation in cancers, focusing on recent data on CSCs and making comparisons with adult stem cells.

### Epigenetic regulation in germline stem cells (GSCs)

Germ cells are a unique cell type because they are able to generate an entire organism upon fertilization [26]. Because germ cells are responsible for initiating the next generations, it is crucial that they retain accurate genetic and epigenetic information and properly transmit such information across generations [27]. In many organisms, GSCs initiate a tightly controlled cellular differentiation process called gametogenesis to produce gametes. Like other adult stem cells, GSCs are capable of both self-renewal and differentiation. In addition to extensive knowledge about the role of extrinsic signaling pathways in maintaining GSCs [28], recent studies have shown that epigenetic mechanisms control the decision of GSC self-renewal versus differentiation [29,30].

**Figure 1:**
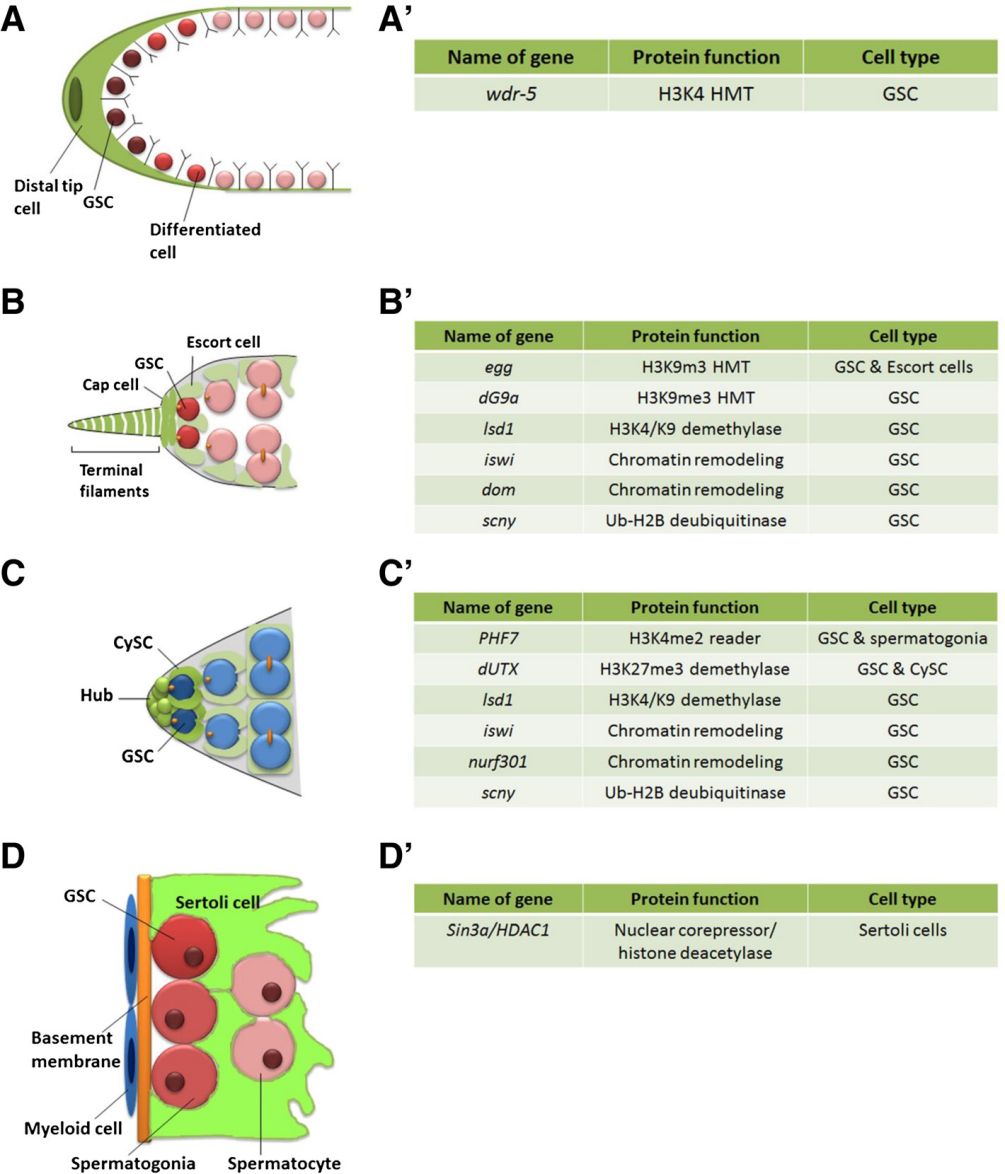
**Illustrations of the GSC niches in *C. elegans, *male and female *Drosophila *and mouse. (A) ***C. elegans* GSC niche. Illustration shows the distal tip cell which acts as a niche to maintain GSCs. Dark red GSCs are within the influence of the niche and are maintained as GSCs. The lighter GSCs are outside the influence of the niche which causes them to differentiate. **(A’)** Summary of epigenetic factors that regulate the *C. elegans* GSC niche. **(B)***Drosophila* female GSC niche. Illustration shows tip of the germarium with GSCs (dark pink, average 2-3) in the niche comprised of terminal filaments and cap cells (dark green). Escort cells are shown in light green. GSC progenies are shown in light pink. **(B’)** Summary of epigenetic factors that regulate the *Drosophila* female GSC niche. **(C)***Drosophila* male GSC niche. Illustration shows tip of the testis with GSCs (dark blue, average 9-12; only 2 are shown here) in the niche comprised of hub cells and CySCs (dark green). Cyst cells are shown in light green. GSC progenies are shown in light blue. Round orange structures represent spectrosomes, and branched orange structures represent fusomes. **(C’)** Summary of epigenetic factors that regulate the *Drosophila* male GSC niche. **(D)** Mouse GSC niche. Illustration shows Sertoli cells which function as a niche to maintain GSCs. Myeloid cells and the basal membrane function as support cells to the niche. GSCs (dark red) differentiate to form spermatogonia (light red) which further differentiate to spermatocytes (pink). **(D’)** Summary of epigenetic factors that regulate the mouse GSC niche.

Histone modifications play an essential role in intrinsically regulating GSC identity and activity. Recent studies have identified a cohort of enzymes called “epigenetic writers” and “epigenetic erasers” that generate or remove a particular histone modification [31,32]. These enzymes are shown to be important for stem cell activities. For example, members of the ASH-2 complex in *C. elegans* act as “epigenetic writers” to generate the active trimethylation of histone H3 lysine 4 (H3K4me3). Deficiencies in members of the ASH-2 complex, such as WDR-5 and H3K4 methyltransferase (HMT) SET-2, lead to misregulation of a subset of genes required for worm longevity [33]. Presence of an intact germline was necessary for lifespan regulation by members of the ASH-2 complex, suggesting that the “epigenetic landscape” of germ cells regulates somatic cell fitness. Additionally, mutations in *wdr-5*, whose function is required for ASH-2 complex stability and activity, lead to decreased GSCs and improper gametogenesis, suggesting another role for H3K4 methylation in maintaining GSC identity and proper differentiation [34] (Figure 1A).

HMTs are also required for gametogenesis in *Drosophila melanogaster*. The *Drosophila* male and female GSC lineages are both paradigmatic systems to study adult stem cells in their physiological environment, or niche [35-40]. In females, 2–3 GSCs reside in the germarium located at the tip of each ovariole [41], and each ovary contains about 16 ovarioles. Within the female GSC niche, GSCs directly associate with somatic cells (i.e., cap cells, terminal filaments, and escort cells, Figure 1B). GSCs mutant for *eggless (egg),* a HMT that generates the repressive H3K9me3 modification, display both maintenance and differentiation defects [36]. Removal of *egg* function from germ cells using FLP-mediated FRT recombination leads to GSC maintenance defects in the niche, suggesting that Egg is required intrinsically for GSC self-renewal. Loss of *egg* in GSCs leads to decreased expression of bone morphogenetic protein (BMP) pathway components, which are necessary and sufficient for GSC self-renewal. Consistent with the results observed using loss-of-function alleles, knockdown of *egg* using an RNAi transgene leads to GSC loss [36]. However, using another RNAi transgene leads to enlarged germaria due to the accumulation of GSC-like cells, suggesting an intrinsic role for *egg* in regulating GSC differentiation [36,42]. It is rare for a single gene to be required for both GSC maintenance and differentiation. The contradictory results could stem from one or both of the RNAi transgenes used having off targets. Interestingly, loss of *egg* in escort cells in the female GSC niche leads to germaria accumulating GSC-like cells, indicating that Egg is also required non-cell-autonomously for proper differentiation of GSCs. Most of the GSC-like cells away from the niche still express high levels of BMP pathway components, suggesting that Egg acts in escort cells to prevent ectopic BMP signaling and allow proper GSC differentiation. It is remarkable that Egg regulates both GSC self-renewal and differentiation by having an opposite effect on the same signaling pathway in a cell type-specific manner [36].

Another H3K9 methyltransferase in *Drosophila*, dG9a, is required for the formation of functional spectrosome, an organelle required for asymmetric divisions of female GSCs. As a result of spectrosomal dysfunction, germaria mutant for *dG9a* accumulate disorganized germline cysts that fail to specify the oocyte for oogenesis [35].

“Epigenetic erasers” reverse particular histone modifications, which have been shown to regulate adult stem cell maintenance [39,40]. For example, histone demethylases remove methyl groups from methylated lysine residues of histones [43]. The lysine-specific demethylase 1 (Lsd1), which demethylates histone 3 on both lysine 4 and lysine 9 (H3K4/K9), was shown to function in the ovary to prevent GSC tumor formation and maintain proper egg chamber development [39].

In *Drosophila* testis, a group of 8–12 GSCs reside in a niche comprised of two types of somatic cells: hub cell and cyst stem cells (CySCs) (Figure 1C). GSCs undergo asymmetric cell divisions to ensure the balance between self-renewal and differentiation [44]. Recent studies from our group reveal a very interesting phenomenon. Specifically, during GSC asymmetric divisions, preexisting histone 3 (H3) is preferentially retained in the GSC, while newly synthesized H3 is enriched in the other daughter cell called a gonialblast (GB) committed for differentiation. We further demonstrate that both asymmetric H3 segregation during GSC mitosis and post-mitotic rapid turnover of preexisting H3 in GB contribute to this asymmetric H3 distribution. Such asymmetric inheritance of H3 could be a mechanism for the ability of GSC to maintain its unique gene expression profile, as well as allowing GB to reset its chromatin structure for differentiation [45,46]. Interestingly, such an asymmetric H3 distribution pattern is abolished in testicular tumor in which GSCs are overproliferative [45], suggesting that this asymmetric H3 inheritance is related to different cell fates from asymmetric cell divisions. It will be interesting to investigate whether other stem cells use similar mechanisms for a reliable epigenetic inheritance.

Recently, several proteins that generate, recognize, or remove specific histone modifications have been reported to play essential roles in male GSC maintenance. For example, an “epigenetic reader” encoded by the *PHD finger protein 7* (*PHF7*) gene recognizes and associates with the active H3K4me2 mark. PHF7 is highly expressed in early germ cells and is required for GSC maintenance and spermatogonial differentiation [37]. An “epigenetic eraser”, *Drosophila* Ubiquitously transcribed tetratricopeptide repeat gene on the X chromosome (dUTX), is the sole enzyme that demethylates the repressive H3K27me3 mark [47]. Our group found that dUTX regulates testis niche architecture by targeting the Janus kinase signal transducer and activator of transcription (JAK-STAT) signaling pathway, a major pathway required for GSC maintenance [40]. We further showed that dUTX maintains active transcription of an inhibitor of the JAK-STAT pathway encoded by *Suppressor of cytokine signaling at 36E (Socs36E)* gene. Specifically, dUTX removes the repressive H3K27me3 mark near the transcription start site (TSS) of *Socs36E* gene. In addition to its role in maintaining niche architecture, dUTX also functions intrinsically in male GSCs to maintain their adhesion to hub cells by regulating the transcription of *DE-Cadherin*[40]. Interestingly, mammalian UTX, also known as KDM6A, has been shown to regulate reprogramming: *Utx* mutant somatic cells cannot be induced to the ground state of pluripotency [48]. In addition, mutations in the human homolog of UTX cause an increase in H3K27me3 levels and lead to human cancers [49]. These observations suggest that UTX H3K27me3 demethylase maintains stem cell properties in multiple stem cell systems in different species.

Apart from histone modifying enzymes, dynamic regulation by chromatin remodeling factors is also required to maintain GSC activity and identity. Chromatin remodeling enzymes use ATP hydrolysis to alter histone-DNA contacts [50]. In *Drosophila*, nine ATP-dependent remodelers have been classified into four families based on their structural similarities: (1) imitation switch (ISWI) family members which all have a SANT domain, (2) SWI2/SNF2-related proteins which share a bromodomain, (3) CHD family members which all have a chromodomain, and (4) Rad16 family members which possess a ring finger [51]. Interestingly, ISWI maintains GSCs in both males and females, suggesting a common epigenetic mechanism in both sexes [52,53]. ISWI and Nurf301 are two of the four subunits that form the nucleosome remodeling factor (NURF) complex. In male flies, mutations in either *iswi* or *nurf301* lead to decreased GSCs [52]. In females mutant for either *iswi* or a second ATP-dependent remodeling factor known as *Domino (DOM)*, GSCs are lost as a result of premature differentiation [53]. In both sexes, the premature differentiation of GSCs is caused by precocious expression of the *bag of marbles* (*bam*) gene, which is necessary and sufficient for GSC differentiation.

The role of chromatin remodeling factors in maintaining GSC activity is also evident in mammals. In mice, Sertoli cells maintain physical contact with germ cells throughout gametogenesis (Figure 1D). They direct formation of the stem cell niche by coordinating the functions of other support cell populations [54]. SIN3A, a nuclear corepressor that associates with histone deacetylase-1 (HDAC1), is highly expressed in Sertoli cells. HDACs remove acetyl groups from specific lysine residues on histone tails, and their activity is often associated with transcriptional repression. Testes from mice lacking *Sin3a* exhibit a wide range of defects from loss of GSCs and proliferative spermatogonia to failure of spermatid differentiation. GSC markers, such as *Oct4* and *Lin28*, are downregulated in *Sin3a* mutant testes [55,56], suggesting that the chromatin structure of Sertoli cells is essential for maintaining active transcription of key regulators for GSC maintenance [55,56], probably through signaling pathways.

### Epigenetic regulation in intestinal stem cells (ISCs)

**Figure 2:**
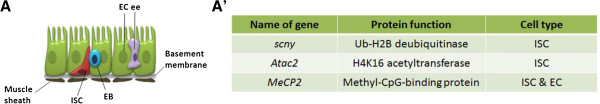
**Illustration of the ISC niche in Drosophila. (A)** Drosophila ISC lineage. Illustration shows an ISC (red) located at the basement membrane. Another daughter cell of ISC is an EB cell (blue), which further differentiates to EC (green) and ee (purple). **(A’)** Summary of epigenetic factors that regulate the Drosophila ISC niche.

The *Drosophila* midgut is the primary organ for food digestion and nutrient absorption. Therefore, its maintenance is essential for organismal growth and survival. The midgut in *Drosophila* comprises an epithelial monolayer that is surrounded by two layers of visceral muscle. Unlike GSCs, ISCs could not be easily identified based on their anatomic locations within the tissue. However, the lineage-tracing technique was utilized to successfully determine that ISCs reside at the basal side, adjacent to the basement membrane of midgut [57,58]. ISCs are multipotent in that they divide asymmetrically to self-renew and give rise to progenitor cells called enteroblasts (EBs). Activated Notch is sufficient for ISCs to differentiate to EBs, while activated Wnt signaling leads to ectopic ISC self-renewal [59,60]. EBs further differentiate into two cell types: absorptive enterocytes (ECs) and enteroendocrine cells (ees) [57,58] (Figure 2). While many studies on ISCs have focused on signaling pathways, such as Notch and Wnt signaling pathways [61], recent studies have uncovered important roles of epigenetic mechanisms in maintaining ISC identity and activity.

Several histone-modifying enzymes have been implicated in maintaining ISCs. One example is the Scrawny (Scny) enzyme that deubiquitinates mono-ubiquitinated H2B and functions in gene silencing. Adult flies mutant for *scny* rapidly lose ISCs due to inappropriate activation of the Notch pathway, which leads to ISC differentiation. Furthermore, *scny* mutant flies have decreased GSCs in testes and ovaries, as well as ISCs, suggesting that a single histone- modifying enzyme is required in multiple stem cell systems [62]. Interestingly, cells mutant for *scny* have elevated ub-H2B and H3K4me3 signals, which probably leads to more open chromatin and active transcription of Notch target genes [62]. Consistent with the requirement of ub-H2B for cellular differentiation, in female GSC lineage, ub-H2B signal is undetectable in GSCs, but detectable in the cystoblasts (CBs), the immediate daughter cells of GSCs committed for differentiation [63]. Recently, a histone acetyltransferase (HAT) encoded by the *Atac2* gene has been shown to regulate the activity of ISCs [64]. HATs transfer acetyl groups to specific lysine residues on histone tails, a modification that is mostly associated with active transcription. Atac2 is a component of the Ada-Two-A-containing (ATAC) complex, which acetylates K16 on H4 [65,66]. Loss of *Atac2* leads to increased ISCs, whereas overexpression of *Atac2* promotes ISC differentiation [64]. The molecular mechanism by which Atac2 regulates ISC differentiation remains unknown, but one possibility is that Atac2 activates Notch target genes by generating the H4K16ac mark at their promoter regions.

In addition to histone-modifying enzymes, dynamic regulation of ISC activities is achieved by DNA modifications. DNA methylation at cytosines is usually associated with repressive gene expression (reviewed in [2]). Mammalian methyl-CpG-binding protein-2 (MeCP2) recognizes methylated DNA and associates with SIN3A and HDAC1 histone-modifying enzymes, acting as a bridging factor between DNA methylation and histone modifications [67]. Unlike mammals, DNA methylation is only detectable in the early stages of *Drosophila* embryos [68]. Interestingly, expression of human MeCP2 (hMeCP2) in *Drosophila* ECs in midgut alters the cytological distribution of heterochromatin protein-1 (HP-1), as determined by immunofluorescence, and stimulates ISC proliferation. These observations suggest that hMeCP2 misregulates genes important for ISC maintenance [69].

### Epigenetic regulation in hair follicle stem cells

**Figure 3:**
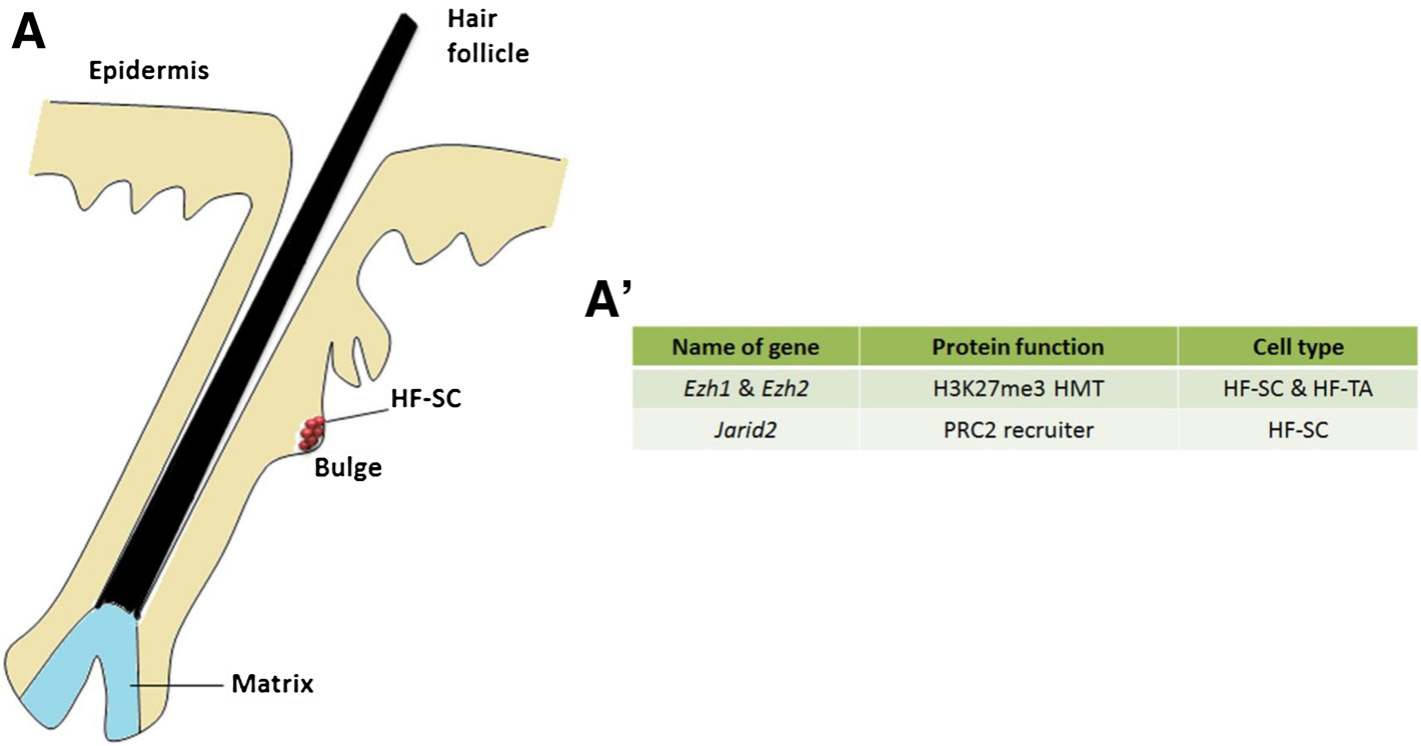
**Illustration of the mammalian HF-SC niche. (A)** Mammalian hair follicle and part of epidermis. Hair follicle stem cells or bulge stem cells reside in the bulge. **(A’)** Summary of epigenetic factors that regulate the HF-SC niche.

In mammals, the stem cells within the hair follicle niche (HF-SCs) are required to sustain hair regeneration and pigmentation in a cyclical manner. HF-SCs refer to both epithelial hair follicle stem cells and melanocyte (i.e., pigment) stem cells, both of which reside at the base of the noncycling hair follicle in the bulge area (Figure 3). Two hallmarks of HF-SCs are their extended state of dormancy and slow cycling, properties which predispose these cells to accumulate genetic mutations and epigenetic aberrations that lead to tumor formation [70]. Remarkably, the proliferation and differentiation cycle of melanocytes is synchronized to the cycle of hair follicle cells in order to regenerate pigmented hair [71]. Hair follicles periodically undergo hair growth (anagen) followed by destruction (catagen) and rest (telogen), during which both stem cell populations remain quiescent for weeks in adulthood.

Several signaling pathways, including Wnt, BMP/TGF-β and mitogen-activated phosphokinase (MAPK) pathways, have been reported to play essential roles in activating both stem cell populations coordinately [72-74] in order to start a new cycle of hair follicle generation. Recent reports have uncovered key roles of specific histone-modifying enzymes in regulating the balance between quiescence and activation of HF-SCs. For example, Polycomb group (PcG) proteins, which are comprised of Polycomb repressive complex 1 (PRC1) and PRC2, have been shown to maintain the cyclical nature of hair follicle regeneration. Using chromatin immunoprecipitation, followed by ChIP-seq, a high-throughput sequencing technique, chromatin changes upon transition from HF-SCs to transit-amplifying progenies (HF-TA) have been characterized. In HF-SCs, PcG represses hair follicle differentiation by generating the repressive H3K27me3 mark at TSSs of key differentiation genes, which are repressed in HF-SCs, but expressed in HF-TAs. Reciprocally, genes required for HF-SC maintenance acquire high levels of H3K27me3 in HF-TA cells, which was found to be necessary for proper HF-TA differentiation [75]. Because PRC2 components *Enhancer of Zeste homolog 1* (*Ezh1*) and *Ezh2* encode H3K27me3 methyltransferases in mice, *Ezh1/2* double knockout HF-SCs have reduced H3K27me3 levels and decreased proliferation. Real-time PCR (RT-PCR) and immunofluorescence analyses in mutant HF-SCs revealed increased transcription of the *Ink4b*/*Ink4a*/*Arf* gene locus, which encodes cell cycle inhibitors p16, p15 and p19 [76]. Increased expression of cell cycle inhibitors may lead to HF-SC proliferation defects.

Another recent study reported the role of Jarid2 in maintaining HF-SCs. Jarid2 is a member of the JumonjiC (JmjC) domain-containing family of proteins. Using ChIP, followed by quantitative PCR (qPCR) in *Jarid2* conditional knockout (cKO) neonatal keratinocytes, H3K27me3 was demonstrated to have reduced levels at PRC2 target genes, suggesting that Jarid2 recruits PRC2 to their targets. These data are consistent with the function of Jarid2 in embryonic stem cells (ESCs) [77,78]. Although Jarid2 has been found to be dispensable for HF-SC establishment and maintenance, in *Jarid2* cKO mice, loss of *Jarid2* leads to increased expression of p16, which results in reduced proliferation and delayed hair follicle cycling of HF-SCs [79].

### Abnormal epigenetic regulation in cancers

Self-renewal and proliferative abilities are essential for maintaining stem cell number and preventing tissue dystrophy. However, several mechanisms are required to tightly regulate stem cell self-renewal and proliferation in order to prevent uncontrolled cell expansion and tumor generation. The cancer stem cell (CSC) model proposes that a subpopulation of tumor cells self-renew and give rise to more differentiated cells that form the tumor [19-24,80]. CSCs are highly proliferative and responsible for sustained tumor growth, as well as new tumor formation upon metastasis [81]. Therefore, understanding the cellular and molecular characteristics of CSCs may have many implications for developing therapeutic strategies against cancers.

Several epigenetic mechanisms have been implicated in maintaining the identity and activity of CSCs (Table 1). For example, global DNA hypomethylation has been shown to be a hallmark of many benign and invasive tumors [82-84]. *S100A4*, a metastasis-associated gene, has been found to be hypomethylated in colon cancer [85], and hypomethylation at the oncogene *R-RAS* region is associated with gastric cancer [86]. DNA demethylation is a recently identified phenomenon with the discovery of the *ten-eleven-translocation* (*TET*) family genes. Members of the Tet family of proteins (Tet1/2/3) are dioxygenases that convert cytosine-5-methylation (5mC) to 5-hydroxymethyl-cytosine (5hmC) [87,88], the removal of which contributes to the DNA demethylation process [89]. Interestingly, levels of 5hmC are substantially reduced in a number of human cancers, including breast, liver, lung and pancreatic cancers, which was found to be associated with dramatically reduced expression of all three *TET* genes [90]. It is very likely that abnormal epigenetic regulation at *TET* genes’ loci leads to their reduced expression. Significant loss of 5hmC is also a feature of human melanomas, and, interestingly, introduction of active *TET2* suppresses melanoma growth [91].

**Table 1:** Summary of epigenetic factors that regulate CSCs

**Name of gene**	**Protein function**	**Cancer type**
*TET* family (*TET1, TET2, TET3*)	DNA demethylases	Prostate cancer, breast cancer, liver cancer, lung cancer, leukemia, melanoma
*Dnmt1*	DNA methylase	Leukemia
*Ezh2*	H3K27me3 HMT	Leukemia, breast cancer, prostate cancer, pancreatic cancer, ovarian cancer
*Bmi1*	PRC1 component	Glioblastoma
*MLL1*	H3K4me3 HMT	Glioblastoma
*Lsd1*	H3K4/K9 demethylase	Teratocarcinoma, embryonic carcinoma, seminoma

On the other hand, genetic mutations in *TET* genes have been found in other cancers, including leukemia and lymphoma [91-94], suggesting an essential role of DNA demethylation in carcinogenesis. Specifically, *TET2* has been shown to act as a critical tumor suppressor and is frequently mutated in leukemia and myeloid cancers [95,96]. *TET1* has also been shown to be a tumor suppressor in various cancers, including prostate and breast cancers [97,98]. Interestingly, while *TET* genes are frequently downregulated in tumors, a recent study reported that *TET1* is upregulated in *MLL-*rearranged leukemia which is accompanied by a global increase in 5hmC levels, suggesting a role for *TET1* as an oncogene instead of a tumor suppressor. Such an observation highlights the importance of tissue context in understanding a gene’s function since *TET1* can act as a tumor suppressor in solid tumors, but as an oncogene in leukemogenesis. Furthermore, while both Tet1 and Tet2 have similar catalytic activities, they play opposing pathological roles in leukemogenesis, probably due to different target genes.

On the other hand, increased DNA methylation has been detected at promoters of tumor suppressor genes, such as *p16* in melanoma [99], *RB1* in retinoblastoma [100], and *RUNX3* in human brain tumors [101]. Hypermethylation was also detected at the promoter region of *Caspase 8 associated protein 2 (CASP8AP2)* gene in acute lymphoblastic leukemia [102]. DNA methylation is generated by DNA methyltransferase 1 (DNMT1) and maintained by DNMT3A and DNMT3B in humans [103-105]. DNA methylation has been shown to regulate CSC activity and tumor growth. For example, cKO of *Dnmt1* in mice with leukemia blocks further development of pre-existing leukemia. Furthermore, halving the level of Dnmt1 in wild-type mice leads to impaired leukemia stem cell self-renewal and survival, probably from hypomethylation and derepression of a number of tumor suppressor genes. Interestingly, using ChIP with H3K27me3 antibodies, the authors found that EZH2-controlled target genes are also derepressed in *Dnmt1* haploinsufficient mice. These data suggest that the PcG complexes might cooperate with DNA methylation to regulate leukemia stem cell activity and tumor growth [106].

Consistent with the role of PcG in deterring tumor development, upregulation of EZH2 leads to aggressive progression of both breast and prostate cancers [107,108]. A recent study reported that a high level of EZH2 expression leads to expansion of breast CSCs. Upregulation of EZH2 may lead to repression of the *RAD51* gene, which is known for DNA double-strand break repair. Failure in DNA repair results in increased genome instability and tumor progression [109]. Furthermore, pharmacological inhibition of PRC2 components, including EZH2, reduces expression of CSC markers and decreases tumor formation and growth in multiple types of cancers [110-112]. Furthermore, knockdown of the oncogene BMI1 reduces expression of glioma stem cell genes and inhibits glioblastoma formation *in vivo*[113]. BMI1 is a component of Polycomb repressive complex 1 (PRC1), which inhibits expression of tumor suppressor proteins *p16* and *p14*. Glioblastoma multiforme (GBM) is one the most common and lethal types of adult brain tumors [114]. Conditions such as hypoxia enhance the expression of glioma stem cell genes. Both hypoxia-inducible factor-1α (HIF1α) and HIF2α are preferentially expressed in glioma stem cells and are required for their maintenance [115-117]. Interestingly, knockdown of mixed-lineage leukemia 1 (MLL1), an H3K4me3 methyltransferase, inhibits expression of HIF2α and reduces glioma stem cell self-renewal and growth [118]. These data suggest that epigenetic regulation of CSCs directly controls cancer initiation and growth. Histone demethylases have also been reported to regulate tumor formation and survival. For example, LSD1, which suppresses gene expression by converting dimethylated H3K4 to monomethylated and unmethylated H3K4, was shown to be highly expressed in pluripotent tumors. Pluripotent tumor cells express pluripotent stem cell markers, such as Oct4 and Sox2, and have the ability to differentiate into many cell types [119-122]. Knockdown of *Lsd1* leads to growth inhibition of pluripotent tumor cells, such as in teratocarcinoma, embryonic carcinoma and seminoma [123].

## Conclusions

In this review, we discussed recent advances in our understanding of epigenetic mechanisms in normal adult stem cell lineages and in tumorigenesis. Several epigenetic mechanisms have been shown to play important roles, including DNA methylation, covalent histone modifications, and chromatin remodeling. Further studies are needed to understand how different epigenetic mechanisms coordinate to ensure normal cellular differentiation in adult stem cell lineages and to prevent cancers. To better understand cancers, researchers are now focusing on the relationship between CSCs and normal stem cells. While both stem cell types have the ability to self-renew and differentiate, adult stem cells require niche cells to maintain their “stemness”, whereas no niche has been identified for any type of CSCs. Additionally, while DNA methylation plays essential roles in tumorigenesis and CSC regulation, little is known about how DNA methylation regulates adult stem cells [68]. Multiple epigenetic factors are now considered targets for therapeutic strategies against cancer, and more studies are needed to elucidate the roles of epigenetic factors in tumor metastasis.

## Competing interests

The authors declare that they have no competing interests.

## Authors’ contributions

LT drafted the manuscript. XC read and edited the manuscript and approved the final manuscript. Both authors read and approved the final manuscript.
